# Structural Characteristics, Electronic Properties, and Coupling Behavior of 12-4-12, 12-3-12, 12-2-12 Cationic Surfactants: A First-Principles Computational Investigation and Experimental Raman Spectroscopy

**DOI:** 10.3390/molecules29122880

**Published:** 2024-06-17

**Authors:** Shiru Lin, Daisy Woodring, Richard D. Sheardy, Nasrin Mirsaleh-Kohan

**Affiliations:** Division of Chemistry and Biochemistry, Texas Woman’s University, Denton, TX 76204, USA; dwoodring@twu.edu (D.W.); rsheardy@twu.edu (R.D.S.)

**Keywords:** surfactant structure, Density Functional Theory (DFT) calculations, Grand Canonical Monte Carlo (GCMC) simulations

## Abstract

In this study, we present a comprehensive first-principles computational investigation focused on the structural characteristics, electronic properties, and coupling integrations of three cationic Gemini surfactants: 12-4-12, 12-3-12, and 12-2-12 ((CH_3_(CH_2_)_11_)(CH_3_)_2_-N^+^-(CH_2_)*_n_*-N^+^(CH_3_(CH_2_)_11_)(CH_3_)_2_, where *n* = 2, 3, or 4). By employing Density Functional Theory (DFT) computations, we aimed to gain insights into the fundamental aspects of these surfactant molecules, and the intermolecular interactions among these surfactant molecules. We examined different conformers of each surfactant, including parallel, wing, and bent conformers, and compared their relative stability and properties. We elucidated that the complex structural characteristics, electronic properties, and molecular arrangements of the surfactants vary according to the number of carbon atoms in the central spacer. We also conducted experimental Raman spectroscopy on the three surfactants to compare the results with our computational findings. Furthermore, we computed the coupling behaviors of different conformers of 12-4-12 surfactants in order to gain insights into their coupling mechanism.

## 1. Introduction

Surfactants, a class of amphiphilic compounds, have significant implications in various industries [1,2,3], pharmaceuticals [4], and everyday consumer products [5,6] due to their unique properties and versatile applications [7,8,9]. These molecules possess a hydrophilic head and a hydrophobic tail, allowing them to reduce surface tension and facilitate the formation of stable interfaces between immiscible phases.

Gemini surfactants, a relatively new class of surfactants, have received significant attention in years [10,11]. They are characterized by a molecular structure consisting of two hydrocarbon chains linked by a spacer [12]. The presence of two hydrophobic tails imparts distinctive properties to Gemini surfactants, making them particularly intriguing for research [13,14] and applications [12,15]. The length and nature of the spacer between the hydrocarbon chains can significantly influence their behavior and functionality [16,17,18].

The investigation of Gemini surfactants and their properties holds great importance. Understanding the intermolecular interactions, self-assembly processes, and structural characteristics of these surfactants is crucial for their effective utilization and design of novel surfactant-based materials. Although traditional experimental methods provide valuable insights, they often have limitations in accessing detailed atomic-level information. Computational chemistry investigations have emerged as powerful tools to overcome these limitations. By employing first-principles calculations [19], we can delve into the intricate details of Gemini surfactants. However, due to the relatively large number of atoms and conformations involved in Gemini surfactants, accurate first-principle calculation studies require high computational demands, resulting in a lack of systematic computational research on Gemini surfactants. Density Functional Theory (DFT) computations have been applied to study the effects of the carbon chain length of Gemini surfactants on the inhibition of the acid–rock reaction rate, showing that the longer carbon chain is more easily overturned on the rock surface. Molecule Dynamics (MD) simulations also indicate that the surfactants with longer carbon chains can aggregate easily on the surface [20]. Though computational studies have explored the effect of chain lengths, the influence of the length of the linker and the properties and behaviors of different conformers remain insufficiently characterized.

In this study, we aim to unravel the effects of varying the number of carbon atoms in the central spacer on the properties of surfactants. By examining the structures and properties of these conformers at the atomic level, we can gain fundamental insights that will enhance our understanding of micellization processes, providing valuable information for future explorations in this area. Specifically, we focus on (CH_3_(CH_2_)_11_)(CH_3_)_2_-N^+^-(CH_2_)*_n_*-N^+^(CH_3_(CH_2_)_11_)(CH_3_)_2_ structures, where *n* = 2, 3, or 4 and, as such, are designated as 12-2-12, 12-3-12, and 12-4-12. By conducting comprehensive first-principles computational investigations, we gained insights into the structural characteristics, electronic properties, and coupling integrations of these surfactants. Our results indicate that the lengths of the spacer in Gemini surfactants influence the favorable conformations and sequential coupling behaviors. This research contributes to optimal spacer length design to achieve desired properties such as critical micelle concentration (CMC), micelle size, and stability.

## 2. Results and Discussion

### 2.1. Structural Characteristics of Three Conformers of 12-2-12, 12-3-12, 12-4-12 Surfactants

The Gemini surfactants studied here (CH_3_(CH_2_)_11_)(CH_3_)_2_-N^+^-(CH_2_)*_n_*-N^+^(CH_3_(CH_2_)_11_)(CH_3_)_2_) have two quaternized ammines (-N^+^-) separated by a linker of two to four carbons (^+^-(CH_2_)*_n_*-, *n* = 2, 3 or 4). Each nitrogen also has two methyl groups ((CH_3_)_2_-) and one dodecyl chain (CH_3_(CH_2_)_11_-). Due to the existence of long tails of 12-*n*-12 surfactants, one surfactant can have many conformers. 

**Figure 1:**
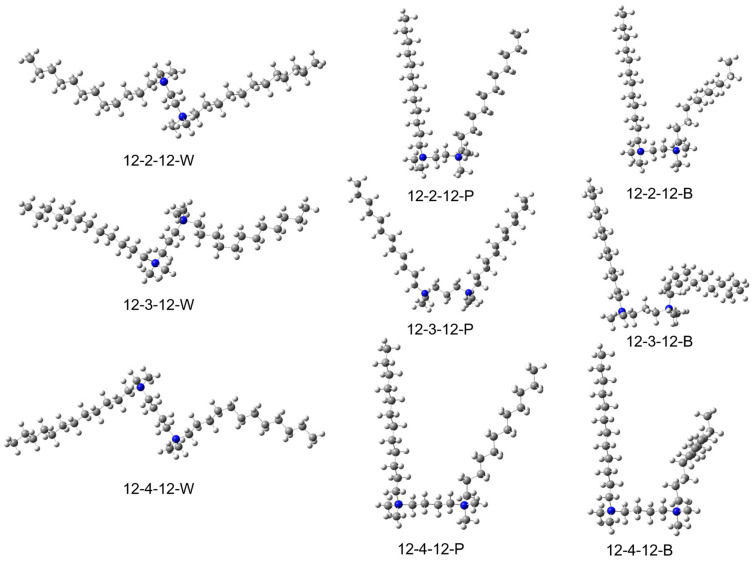
Computational optimized structures of 12-2-12, 12-3-12, and 12-4-12, with wing (W), parallel (P), and bent (B) conformations.

We optimized three different conformers for three different linker lengths (*n* = 2, 3, 4), including wing-shaped (W), parallel-shaped (P), and one straight–bent shape (B) (Figure 1). The two tails extend in opposite directions and away from each other in wing-shaped surfactants (W). Therefore, W presents the largest expansion lengths among three conformers for each pair of 12-n-12 surfactants: 29.88 Å for 12-2-12, 30.92 Å for 12-3-12, 31.93 Å for 12-4-12. The parallel-shaped surfactants (P) have two tails extending in the same direction (Table 1). The tails run parallel to each other, resulting in relatively smaller molecular sizes compared with the other conformers. The longest side of P-surfactants are one tail of 18.36 Å for 12-2-12, the distance of the end of two tails (the longest sides) of 25.14 Å for 12-3-12, one tail of 18.35 Å for 12-4-12 (Table 1). The straight–bent-shaped surfactants (B) features one tail extending in one direction, while the other tail is twisted in the middle, folding in another direction. These results in B surfactants having a size in between P and W surfactants, but very close to that of P because the longest side of B conformers are often the straight tails, so the molecule sizes are closer to that of P (Table 1).

As shown in Table 2, from analyzing the relative energies of optimized 12-2-12, 12-3-12, and 12-4-12 structures in wing (W), parallel (P), and bent (B) conformations, we observed distinct stability patterns among different conformers. For 12-2-12, the W surfactant emerged as the most stable conformer, followed by the P surfactant, with the B conformer being the least stable. However, the energy difference is a modest 2.84 kcal/mol, suggesting a relatively low energy barrier for conformation transitions. In the case of 12-3-12, the P surfactant exhibited the highest stability, followed by the W surfactant, while the B surfactant was the least stable, with a relative large energy difference of 5.93 kcal/mol, nearly double that of 12-2-12 (2.84 kcal/mol). Similar to 12-2-12, for 12-4-12, the W surfactant ranked as the most stable, followed by the P surfactant, and the B surfactant as the least stable, with an energy difference of 3.52 kcal/mol. In summary, the consistently preferred conformations across these surfactants are the W and P conformations. The reason for this could be, first, that the two tails of W surfactants achieve maximum expansion, thereby minimizing steric repulsion in the structure; on the other hand, P surfactants, while maximizing the overlap of the two tails, achieve stability through parallel stacking, maximizing conjugations of C-H bonds. Therefore, structural stability is a delicate balance between minimizing steric repulsion and maximizing conjugations. Bent surfactants lack a clear advantage in either aspect, resulting in relatively lower stability. Additionally, we observed a similar stability pattern in surfactants with an even number (2 and 4) of carbon atoms in the linker, which is different from surfactants with an odd number (3) of carbon atoms in the linker.

Furthermore, when comparing the most stable conformations of surfactants for each linker length, we find that the size of the most stable conformations for 12-2-12 (W conformer) is 29.88 Å, for 12-3-12 (P conformer) is 25.14 Å, and for 12-4-12 (W conformer) is 31.93 Å. The trend in sizes is 12-4-12 > 12-2-12 > 12-3-12, with the size trend of 12-4-12 and 12-2-12 being more pronounced than that of 12-3-12. This correlates to some extent with our experimental results, where the size order of micelles of 12-3-12 is the smallest compared with 12-4-12 and 12-2-12 [21].

### 2.2. Electronic Properties and Raman of Three Conformers of 12-2-12, 12-3-12, 12-4-12 Surfactants

**Figure 2:**
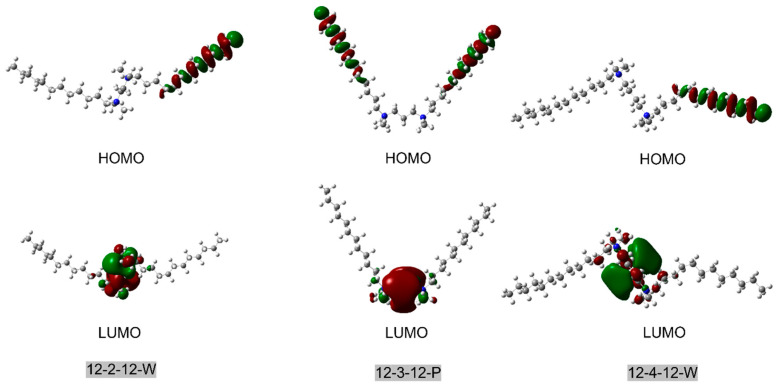
HOMO and LUMO orbitals 12-2-12 W, 12-3-12 P, and 12-4-12 W conformations, where the isovalue was set as 0.02.

We also computed the Highest Occupied Molecular Orbital (HOMO) and Lowest Unoccupied Molecular Orbital (LUMO) of nine surfactant conformers (Figure 2 and Figure S1 (Supplementary Materials)). We found that only in the parallel structure of the surfactant (such as 12-3-12 P in Figure 2) are the HOMO orbitals uniformly distributed between the end of two tails. In the wing-shaped surfactant, where the structure is stretched out, and in the bent-shaped surfactant, where one tail is bent, the HOMO is primarily localized on one tail or the straight tail. This may be because only the tails of the parallel surfactant have high conjugation, allowing the HOMO to be distributed across both tails. This also aligns with our earlier discussion that high conjugation is an important factor contributing to the stability of the parallel structure.

Meanwhile, the LUMO of different conformers is entirely concentrated around the two nitrogen (-N^+^-) atoms of the linker for all structures, representing the active sites of surfactants toward chemical reactions. This is mainly attributed to the positive charge distributed on the nitrogen of the linker of surfactants.

The HOMO-LUMO gap values of different conformers do not differ significantly (Table 3). Among them, the 12-3-12 conformers exhibit the largest difference in HOMO-LUMO gap values, with the maximum being 6.865 kcal/mol, occurring between the 12-3-12-P and 12-3-12-B conformers. All calculated surfactants and conformers display relatively large HOMO-LUMO gaps (>98.574 kcal/mol), indicating that they show insulator properties.

**Figure 3:**
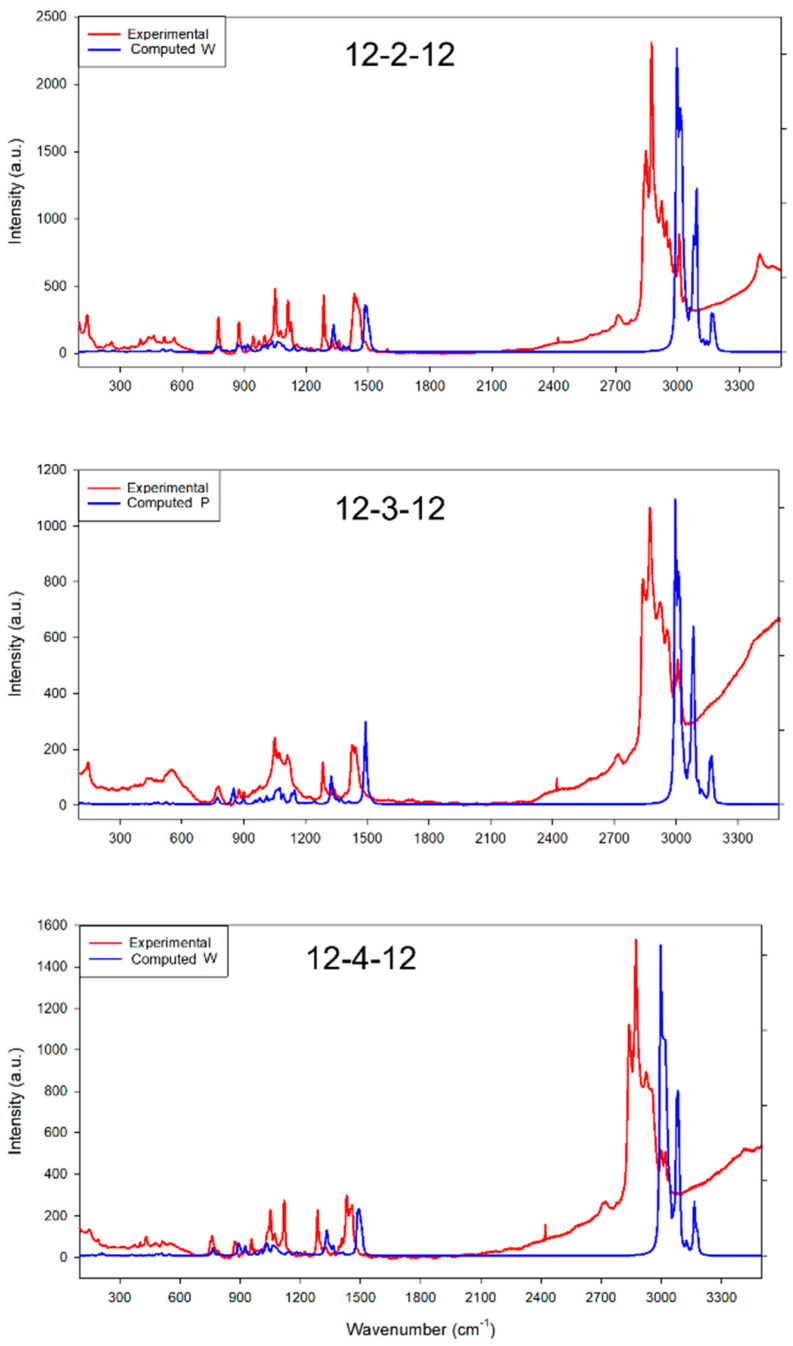
Comparison of experimental Raman spectra for 12-2-12, 12-3-12, and 12-4-12 surfactants and computational Raman spectra for 12-2-12 W, 12-3-12 P, and 12-4-12 W conformations.

Furthermore, we computed the Raman spectra for all three conformations of 12-2-12, 12-3-12, and 12-4-12 to provide a comprehensive comparison with their respective experimental spectra (Figure 3 and Figure S2). The computational Raman values exhibit a slight redshift compared to the experimental values. In the wavenumber range of 1200 to 1600 cm^−1^, the calculated values for the two characteristic peaks of all three surfactants closely match the experimental results. Simultaneously, the calculated values for the three peaks in the 2700–3300 range also align well with the experimental findings. In the 900–1200 cm^−1^ range, the experimental results reveal a distinct presence of small peaks, which is close to what can be found in the computed Raman Spectra for P conformations. However, other conformers also exhibit some small peaks in this range. Due to the very close conformation energies of W, P, and B conformations, the experimental system likely involves a mixture of all three conformations, and, potentially, some dimerizations of three conformers. This complexity is reflected in the fact that it is challenging to directly confirm the presence of individual conformers through Raman spectra.

### 2.3. Coupling Interactions of Different Conformers of 12-4-12 Surfactants

One of the primary focuses of our investigation was the coupling integrations of surfactant structures. For most of the experimental results reporting Gemini surfactants with linkers of four or more atoms, we examined the interactions between the 12-4-12 surfactant molecules to investigate how different conformers form aggregates in various environments.

**Figure 4:**
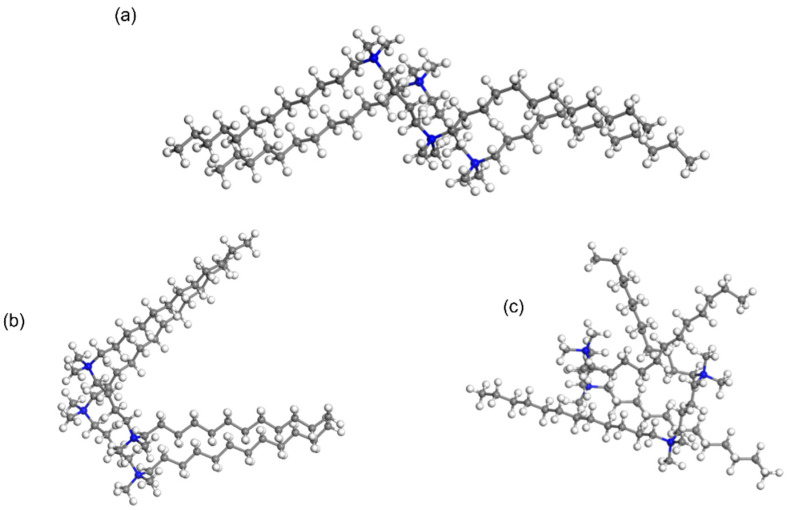
Geometries of paired 12-4-12 with wing (**a**), parallel (**b**), and bent (**c**) conformations computed by the Sorption module.

We employed a forcefield-based Grand Canonical Monte Carlo (GCMC) method to simulate the dynamic behavior of surfactants. Using the fix-loading approach, we immobilized one surfactant molecule while manipulating another structurally identical surfactant to explore the most favorable adsorption geometries of the paired surfactants. This approach allowed us to discern the pairing modes of the two surfactants. Notably, for the wing-shaped surfactant, the most stable pairing involved two molecules arranged side by side in a parallel fashion, which maximizes the conjugation between C-H bonds (Figure 4). Confirming a similar coupling behavior in the parallel-shaped surfactant, we observed a preference for parallel stacking of the two P-surfactant molecules, with the linker and tails aligning in the same direction.

In contrast, the bent-shape surfactants exhibited an entirely distinct coupling behavior (Figure 4). In the case of B-shape surfactants, two surfactants exhibited an opposing orientation, embedding into the space between each other’s tails. This unique conformation resulted in a mutual insertion of the two surfactants, effectively reducing the overall occupied volume. The distinct adsorption patterns of W-shape, P-shape, and B-shape surfactants provide valuable insights into the coupling behavior of these molecules. 

Moreover, we calculated that the average interaction energy for the pairing of wing (W) surfactants is 13.535 kcal/mol, for P surfactants is 14.860 kcal/mol, and for B surfactants is 17.345 kcal/mol, following the order of B > P > W. Considering that the pairing interactions of 12-4-12 (B > P > W) and the order of conformer relative energy (W > P > B) is the opposite, when surfactants aggregate, the energy differences between different conformers become smaller, almost being eliminated. The results not only shed light on the diverse pairing tendencies of these structurally different surfactants, but also hint at their potential to form aggregates with unique structural and functional properties.

## 3. Methods

### 3.1. Computational Methods

The computations were carried out by Becke, 3-parameter, Lee–Yang–Parr (B3LYP) functional [22], and the triple basis set 6-311+G (d,p) [23] in the Gaussian 16 program [4]. All geometric parameters were optimized with no atoms frozen. The frequencies of the molecules were computed after optimization, and no imaginary modes were found for any structures, confirming that all structures were local minima and optimized.

Grand Canonical Monte Carlo (GCMC) simulations [23,24,25,26] in the Sorption module of Materials Studio 2019 [27] were conducted to evaluate the absorption behavior of Gemini surfactants. GCMC is a statistical–mechanical approach, in which the adsorption process is investigated relying on random sampling and probabilistic interpretation in the sorbent framework. We included one Gemini surfactant in the unit cell to calculate the average loading (/mol) and average interaction energy (kcal/mol) among surfactants with the same geometries, where more substantial loading and higher interaction energy indicate stronger interactions among them. The computations were equilibrated for 100,000 GCMC steps, and data were collected for another 1,000,000 production steps to obtain the average amount adsorbed. Fixed loading computations were carried out to determine the most stable interaction geometries of a pair of surfactants with the same structure. All GCMC simulations were carried out at a temperature of 298 K and a fixed pressure of 101.33 kPa with the Metropolis Monte Carlo method [23] and COMPASS (Condensed-phase Optimized Molecular Potentials for Atomistic Simulation Studies) forcefield [28,29]. 

### 3.2. Experimental Methods

The Gemini surfactants were kindly provided by Dr. Steven Bachofer.

Two spectroscopic techniques, Raman and FTIR spectroscopy, were employed to analyze the surfactant samples.

All Raman spectra were obtained using a Thermo Scientific DXR Raman Microscope (Thermo Fisher Scientific, Waltham, MA, USA). The laser wavelength employed was 532 nm with a power of 2.0 mW. The aperture was a 50 µm slit. Surfactant samples were placed on a glass microscope slide and analyzed without any modification.

The FTIR spectra were obtained using a Thermo Scientific Nicolet iS50 FT-IR Spectrometer (Thermo Fisher Scientific, Waltham, MA, USA). The optical velocity was set to 0.4747. The resolution was 4.000. The sample was placed on the ATR stage and analyzed directly. 

## 4. Conclusions

Our first-principles computational investigation provides a detailed understanding of the structural characteristics, electronic properties, and coupling behavior of 12-4-12, 12-3-12, and 12-2-12 surfactants through a combination of computations and experimental verifications. The study explored three conformations of each surfactant, and we found that 12-2-12-W, 12-3-12-P, and 12-4-12-W are the most stable conformations for each surfactant based on optimized energy. However, the conformation energies difference of W, P, and B conformations are rather small; therefore, the experimental system likely involves a mixture of all three conformations, and even some dimerization of three conformers. The comparison of Raman spectra between experimental and computational results further corroborates this conclusion. 

We also applied GCMC simulations to reveal subtle differences in stability and pairing tendencies among different conformers, shedding light on the factors influencing their assembly behavior. Overall, this study investigates the structural, electronic, and pairing behavior of Gemini surfactants with different spacer lengths and different conformers from the atomic level, laying the groundwork for both theoretical and experimental research in this area.

## Figures and Tables

**Table 1:** Lengths of the longest sides of 12-2-12, 12-3-12, and 12-4-12, with wing (W), parallel (P), and bent (B) conformations; unit is Å.

Surfactant	Wing Conformation	Parallel Conformation	Bent Conformation
12-2-12	29.88	18.36	18.56
12-3-12	30.92	25.14	26.17
12-4-12	31.93	18.35	18.35

**Table 2:** The relative energy of 12-2-12, 12-3-12, and 12-4-12, with wing (W), parallel (P), and bent (B) conformations, where the lowest energy of each conformer was set as 0; unit is kcal/mol.

Surfactant	Wing Conformation	Parallel Conformation	Bent Conformation
12-2-12	0	1.38	2.84
12-3-12	3.47	0	5.93
12-4-12	0	1.04	3.52

**Table 3:** The HOMO-LUMO gaps of 12-2-12, 12-3-12, and 12-4-12, with wing (W), parallel (P), and bent (B) conformations; unit is kcal/mol.

Surfactant	Wing Conformation	Parallel Conformation	Bent Conformation
12-2-12	99.340	98.574	99.014
12-3-12	105.640	99.754	106.619
12-4-12	110.685	109.016	110.660

## Data Availability

Please find the optimized surfactant structures on https://github.com/shiru-twu/Cationic-Surfactants.git.

## Supplementary Materials

The following supporting information can be downloaded at: https://www.mdpi.com/article/10.3390/molecules29122880/s1, Figure S1: Highest Occupied Molecular (HOMO, left hand side) and Lowest Unoccupied Molecular (LUMO, right hand side) orbitals of 12-2-12 P, 12-2-12 B, 12-3-12 W, 12-3-12 B, and 12-4-12 P, 12-4-12 B conformers; Figure S2: Comparison of Experimental Raman Spectra for 12-2-12, 12-3-12, and 12-4-12 Surfactants and Computational Raman Spectra for 12-2-12 P, 12-2-12 B, 12-3-12 W, 12-3-12 B, 12-4-12 P and 12-4-12 B conformers.
