# Supplementary material for: Structural Characteristics, Electronic Properties, and Coupling Behavior of 12-4-12, 12-3-12, 12-2-12 Cationic Surfactants: A First-Principles Computational Investigation and Experimental Raman Spectroscopy
Source: Molecules. 2024 Jun 17;29(12):2880. doi: 10.3390/molecules29122880 (PMC11206980; doi:10.3390/molecules29122880)
Supplement: Supplementary file 1 [file molecules-29-02880-s001.zip › molecules-3037690-supplementary.pdf]

Supplementary Materials

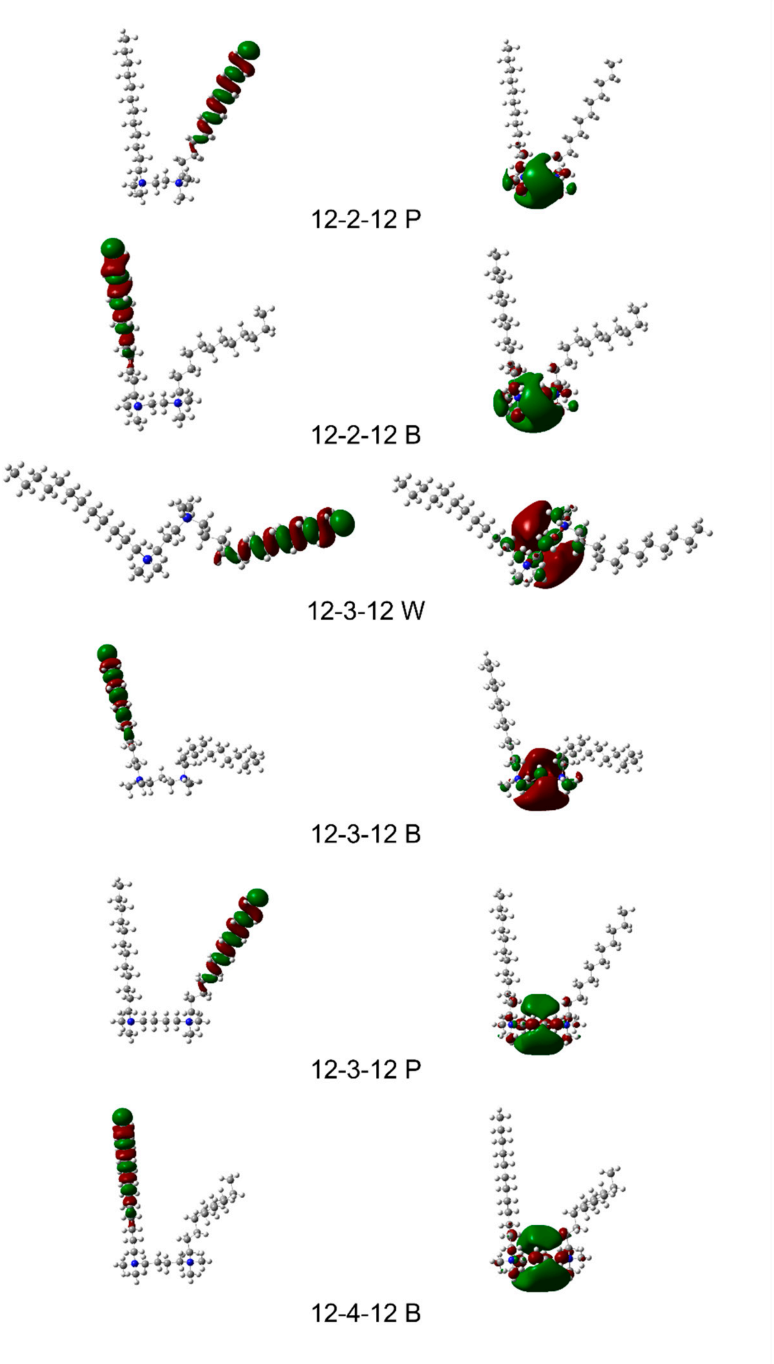

Figure S1. Highest Occupied Molecular (HOMO, left hand side) and Lowest Unoccupied Molecular (LUMO, right hand side) orbitals of 12-2-12 P, 12-2-12 B, 12-3-12 W, 12-3-12 B, and 12-4-12 P, 12-4-12 B conformers.

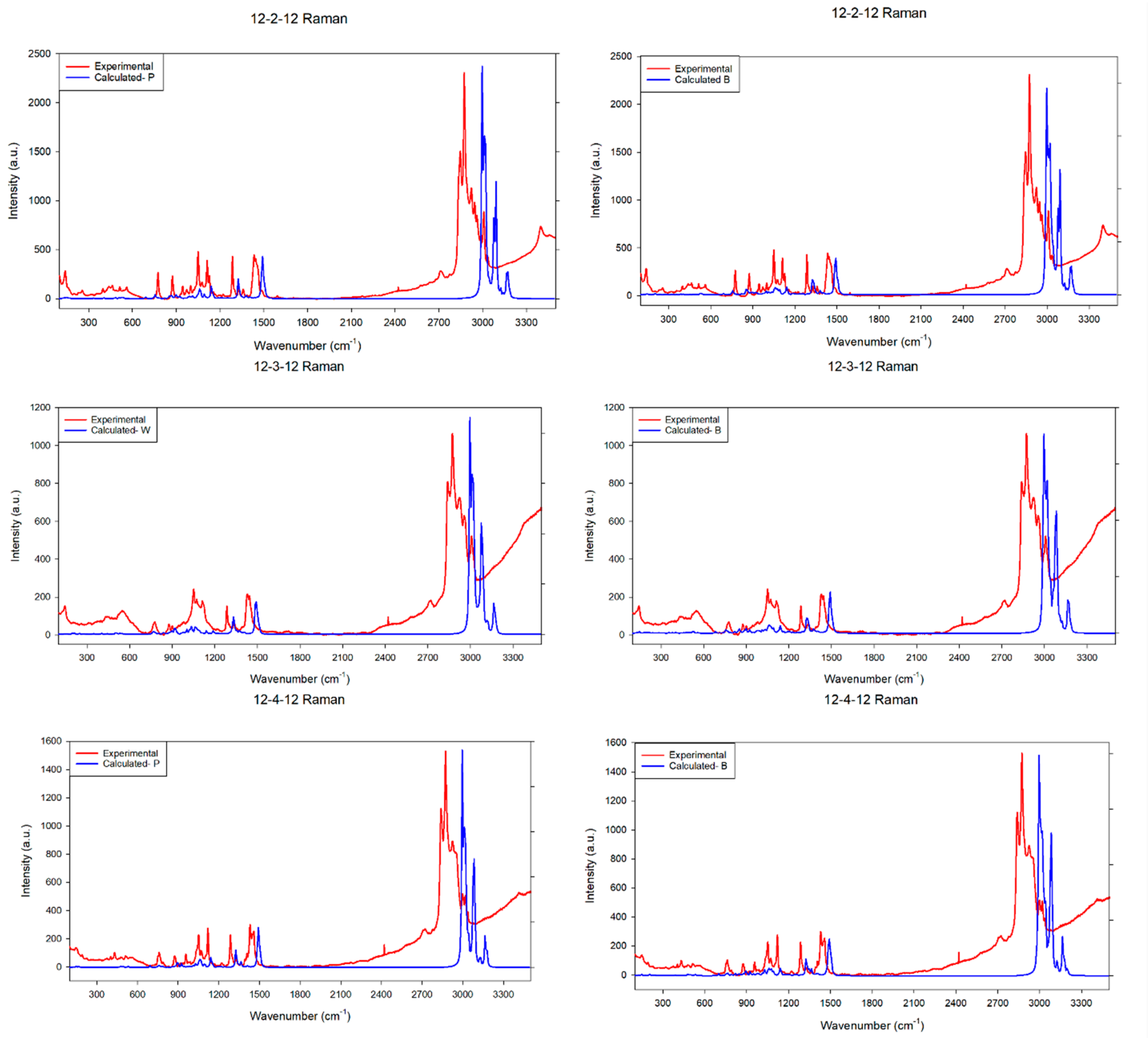

Figure S2. Comparison of Experimental Raman Spectra for 12-2-12, 12-3-12, and 12-4-12 Surfactants and Computational Raman Spectra for 12-2-12 P, 12-2-12 B, 12-3-12 W, 12-3-12 B, 12-4-12 P and 12-4-12 B conformers.
